# Supplementary material for: Robust lasing modes in coupled colloidal quantum dot microdisk pairs using a non-Hermitian exceptional point
Source: Nat Commun. 2019 Feb 4;10:561. doi: 10.1038/s41467-019-08432-6 (PMC6362135; doi:10.1038/s41467-019-08432-6)
Supplement: Supplementary file 1 — Supplementary Information [file 41467_2019_8432_MOESM1_ESM.pdf]

-Supplementary Information –

Robust lasing modes in coupled colloidal quantum dot microdisk pairs using a non-Hermitian exceptional point

Evan Lafalce[1*], Qingji Zeng[1*], Chun Hao Lin[2], Marcus J. Smith[2,3], Sidney T. Malak[2], Jaehan Jung[2,4], Young Jun Yoon[2], Zhiqun Lin[2], Vladimir V. Tsukruk[2], Z. Valy Vardeny[1]

* These authors contributed equally to this work.

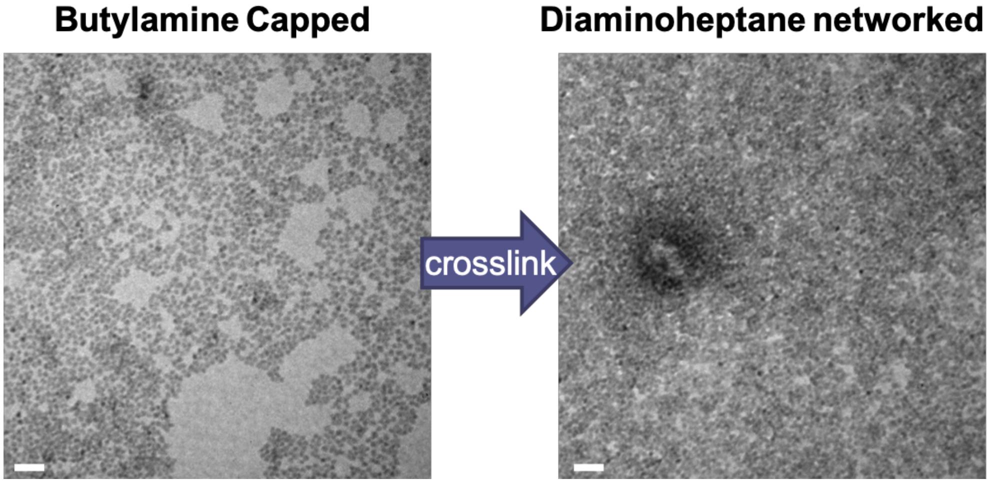

Supplementary Figure 1. **Diaminoheptane networking of quantum dots**

Transmission electrom microscope images of Butylamine capped quantum dots before and after crosslinking with diaminoheptane. Scale bars are 30nm.

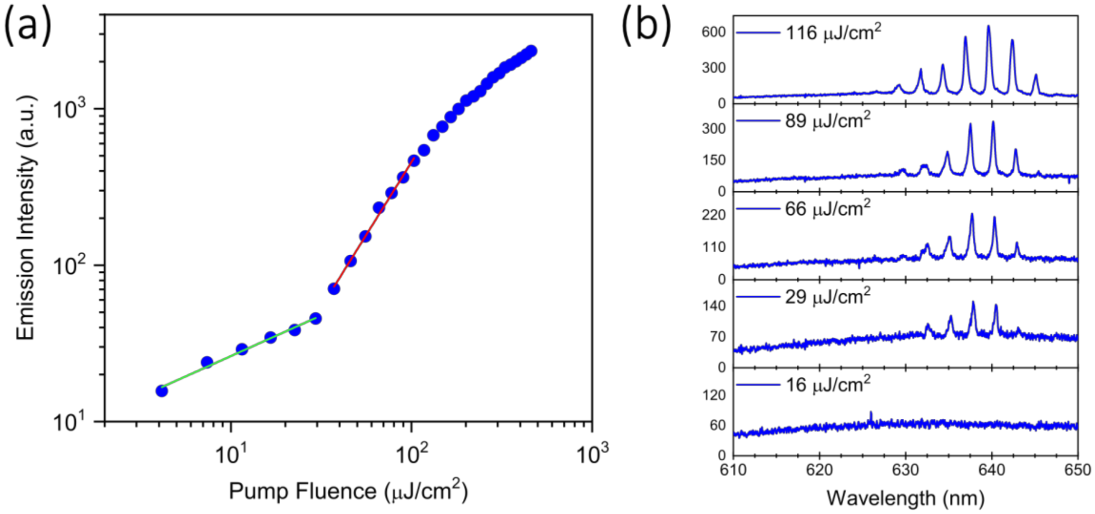

Supplementary Figure 2. **Threshold behavior for individual microdisk lasers**

(a) Light-light curve for an isolated microdisk. The green line indicates the region of sub-linear increase where only spontaneous emission is observed. The red line indicates the super-linear increase above threshold that corresponds to the emergence of cavity modes. (b) Emission spectra at different levels of pump fluence. The cavity modes become apparent at a fluence of 29 $\mu J/cm^2$.

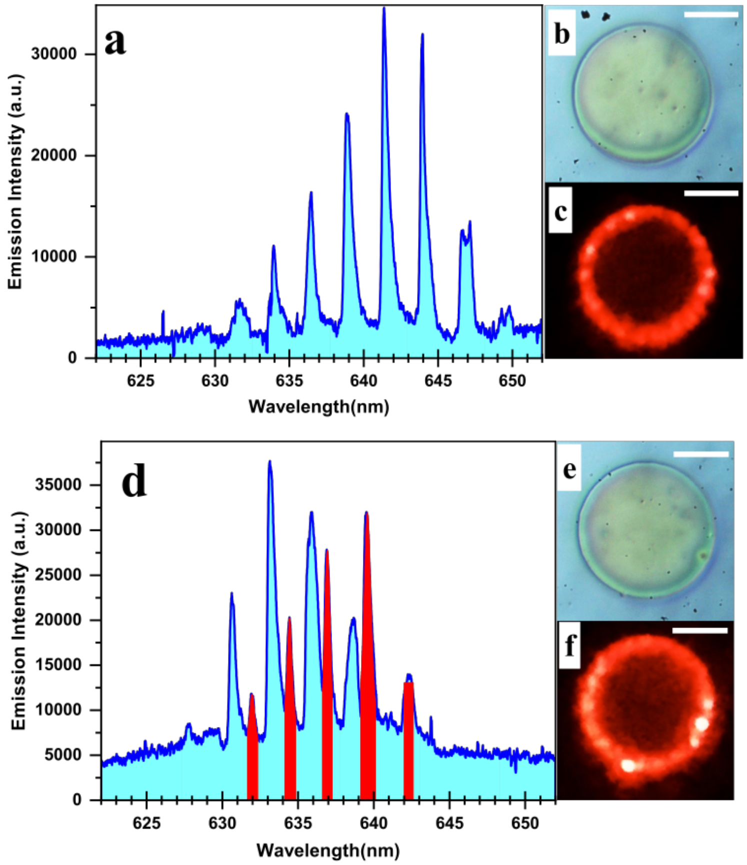

Supplementary Figure 3. **Comparison of disk lacking defects and disk with defects**

a, Emission spectrum, **b**, optical microscope image, and **c,** fluorescent image under lasing conditions from a microdisk without defects. **d**, Emission spectrum, **e**, optical microscope image, and **f,** fluorescent image under lasing conditions from a microdisk with defects. The red shaded regions in **(d)** are Lorentzian fits to the laser modes, emphasizing the two sets of modes. The scale bars in (**b**), (**c**), (**e**), and (**f**) are 10 µm.

The laser emission spectrum in Supplementary Fig. 3a shows a single mode progression, while the fluorescent and bright field microscope images in Supplementary Fig. 3b and 3c, respectively, show a relatively uniform circumference. On the contrary, in Supplementary Fig. 3d, mode splitting is observed in the laser emission spectrum and the fluorescent and bright field

microscope images in Supplementary Fig. 3e and 3f, respectively, show defects that act as mode scattering sites along the circumference.

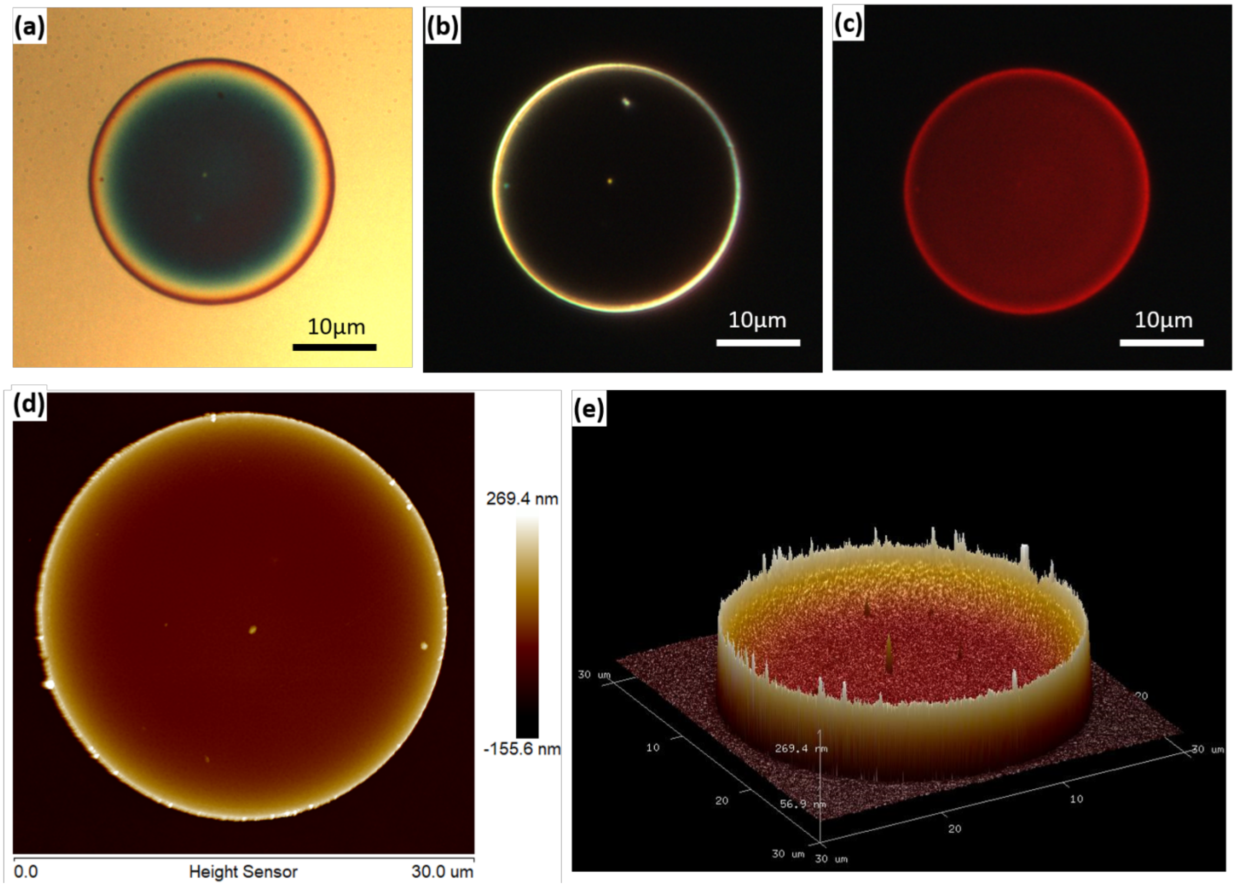

Supplementary Figure 4 Images of individual microdisk

(a) Bright field, (b) dark field, and (c) photoluminescence microscopic imaging of microdisk with diameter of 25 μm. (d) AFM topographical image (top-view) and (e) 3D projection of microdisk with defects formed near circumference.

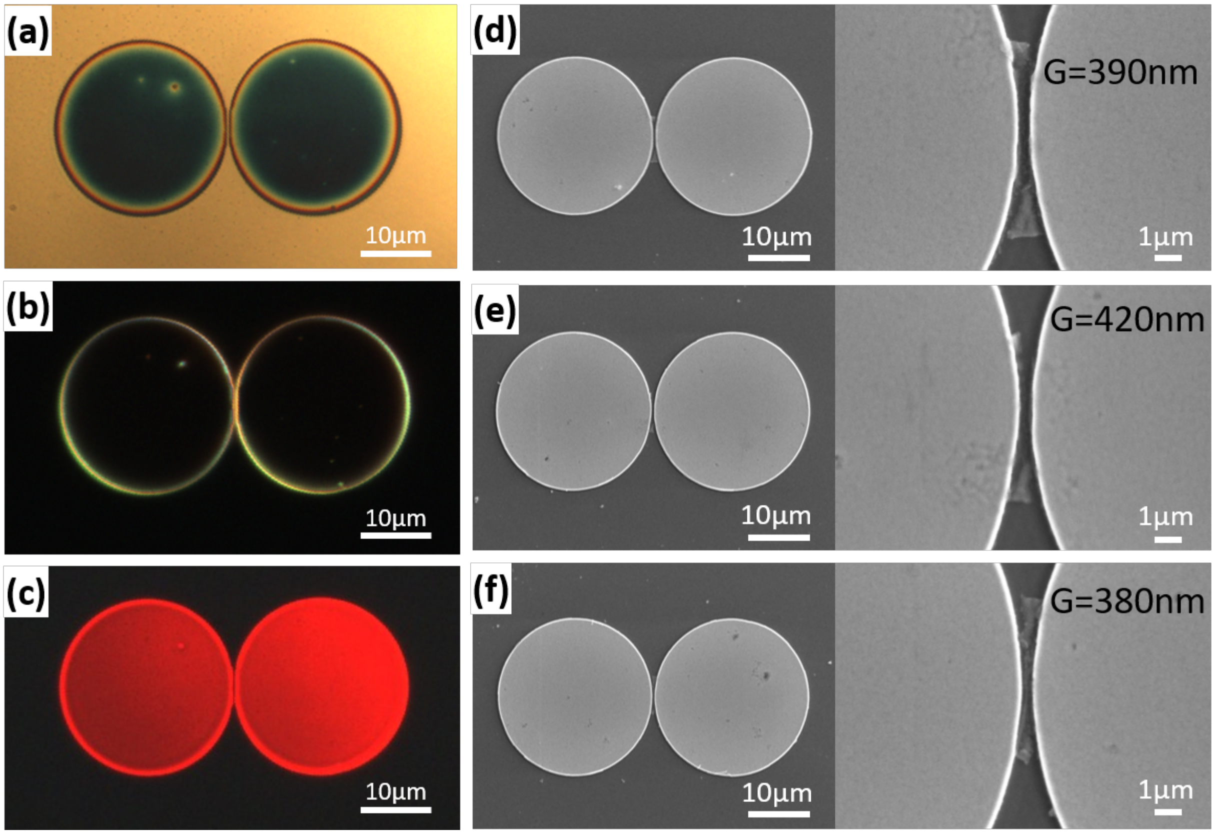

Supplementary Figure 5 Images of microdisk pairs

(a) bright field, (b) dark field, and (c) photoluminescence microscopic imaging of coupled microdisks with disk diameter of 25 μm. (d), (e), (f) SEM images of 3 sets of coupled microdisks.

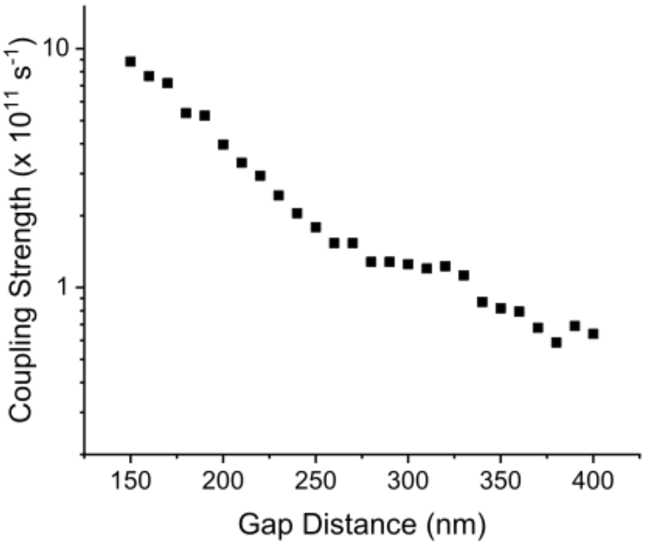

Supplementary Figure 6 Simulated coupling strength vs. distance between microdisks

The coupling rates between microdisks with diameter D = 25μm having refractive index n=1.9 were extracted from FDTD simulations for WGM at 630 nm (in vacuum).

We performed finite difference time domain (FDTD) simulations using the LUMERICAL software package to investigate coupling between WGM modes in our microdisks, as a function of the gap spacing. The theoretical coupling value in our experimental range of interest is ~7 x 10^{10} s^{-1}. Additional experimental-based evidence of coupling between microdisks is provided Supplementary Fig. 15 that suggests the coupling is larger than the theoretically derived values.

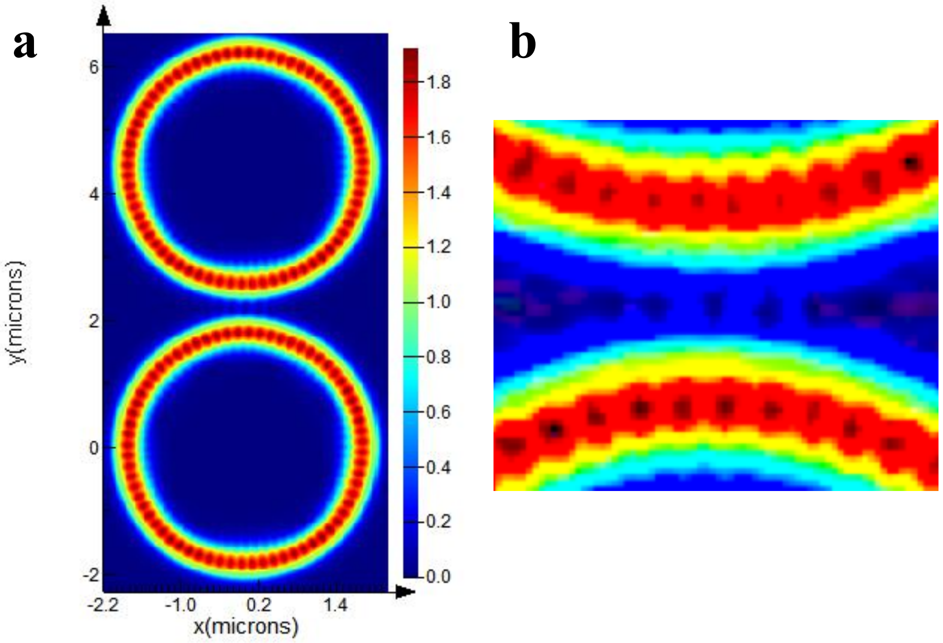

Supplementary Figure 7 Simulated electric field profile in coupled microdisks

a Electric field profiles from the FDTD simulation of two microdisks with diameter *D* = 4μm separated by a distance of 400nm. **b** Close up view of the region between the microdisks in (**a**) emphasizing the overlap of WGM anti-nodes.

FDTD simulations have also shown overlap of WGM modes in disks separated by 400nm. While we have used a realistic wavelength (630nm) and the experimentally determined index of our system (*n* = 1.9), we have used smaller microdisks in the simulation, namely, *D* = 4μm instead of the experimental *D* = 26μm, so that the mode profiles are visible. In the experimental system, the reduced curvature would increase the region of modal overlap providing enhanced coupling between the WGMs on two microdisks[1]. On the other hand, the increased curvature for the simulated system with *D* = 4μm will also increase the bending loss which will increase evanescent interaction and enhance coupling compared to the experimental system.

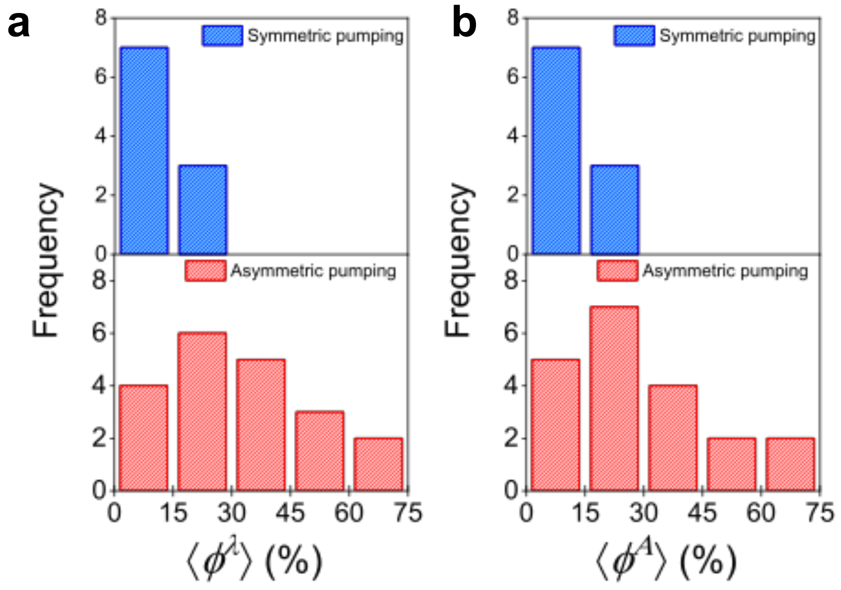

Supplementary Figure 8. Spectrally averaged modal splitting parameters for microdisk pairs.

Frequency distribution of the spectrally averaged modal splitting parameters, **a** $\langle\phi^{\lambda}\rangle$ and **b** $\langle\phi^{A}\rangle$ for microdisks pairs. The splitting parameter from the symmetrically pumped pair is shown in blue while while that for asymmetric pumping of one disk of the pair is shown in red.

To facilitate a quantitative comparison of spectra over a large sample of pairs, we define an empirical parameter to characterize the mode-splitting between any broken-degeneracy WGM mode-pair, based on the wavelength splitting, ϕ_i^{λ}

$$\phi^{\lambda} = \frac{2|\lambda_1 - \lambda_2|}{\Delta\lambda} \qquad (1)$$

Where λ_1 and λ_2 the peak wavelengths of the two modes. The wavelength splitting is normalized by half the free spectral range $\Delta\lambda$, so that together with the amplitude condition we have $0 < \phi < 1$. The maximal observable mode-splitting is thus considered to be $\phi = 1$. Alternatively, we can define the mode splitting in terms of the ratio of amplitudes of the two modes, ϕ_i^{A}

$$\phi^{A} = \frac{A_1}{A_2} \qquad (2)$$

Where A_1 and A_2 are the amplitudes, and the choice is made such that $A_1 < A_2$. The splitting varies for different modes across the spectrum because of the random distribution of the number, size, and relative position. To characterize the effect across the whole spectrum, we take an average of the mode-splitting parameters for each mode-pair

$$\langle\phi\rangle = \sum_i^N \phi_i \frac{1}{N} \qquad (3)$$

where the index runs over the N observable mode-pairs in the spectrum (or equivalently, each WGM azimuthal mode order).

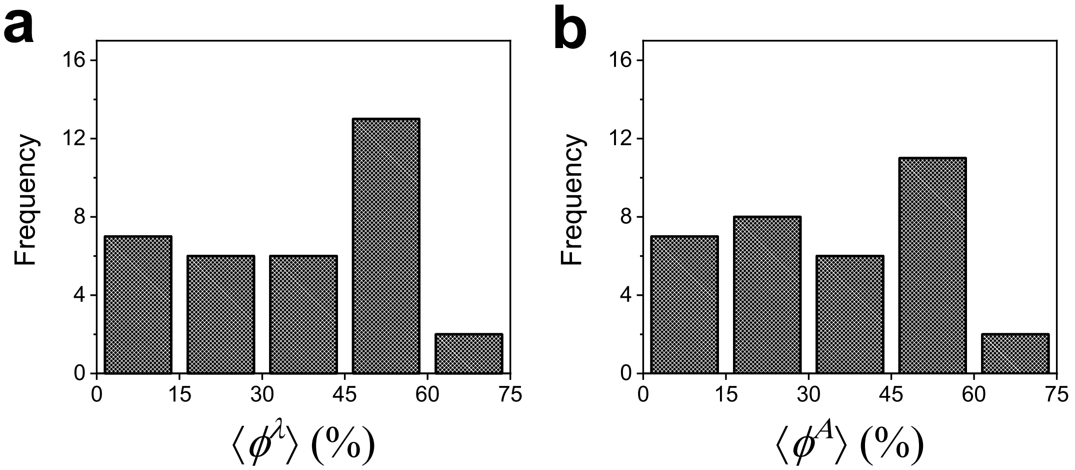

Supplementary Figure 9 Spectrally averaged modal splitting parameters for individual microdisks

Frequency distribution of the spectrally averaged modal splitting parameters **a** $\langle\phi^{\lambda}\rangle$ and **b** $\langle\phi^{A}\rangle$ for individual microdisks.

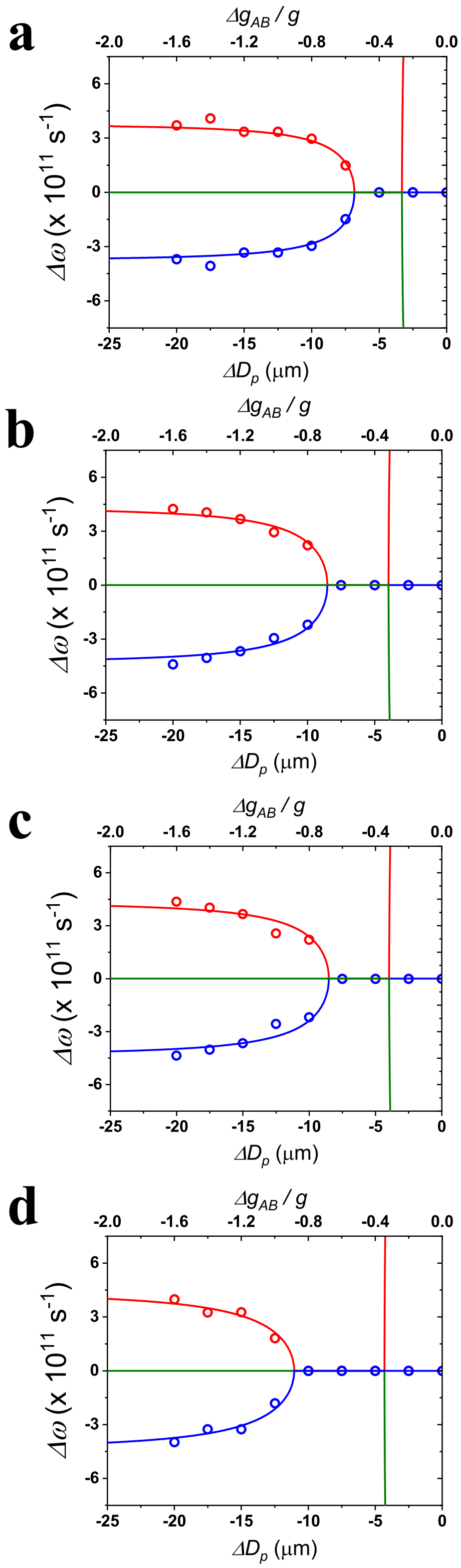

Supplementary Figure 10. **Comparison of the experimental peak positions from Fig. 4a to the model simulations**

The experimental peak positions of the modes (circles) are plotted vs. ΔD_p while the calculated eigenfrequencies (solid lines) are plotted vs Δg_{AB} in units of the total gain g for **a** the mode pair centered at 636.0 nm with model parameters κ = 3.8 x 10^{11} $s^{-1}$and γ_{23} = 1.2 x 10^{12} s^{-1}, **b** the mode pair centered at 638.5 nm with model parameters κ = 4.3 x 10^{11} $s^{-1}$and γ_{23} = 1.4 x 10^{12} s^{-1}, **c** the mode pair centered at 641.0 nm with model parameters κ = 4.3 x 10^{11} $s^{-1}$and γ_{23} = 1.4 x 10^{12} s^{-1}, and **d** the mode pair centered at 643.6 nm with model parameters κ = 3.9 x 10^{11} $s^{-1}$and γ_{23} = 1.6 x 10^{12} s^{-1}. In all cases γ_{13} = 0 s^{-1}.

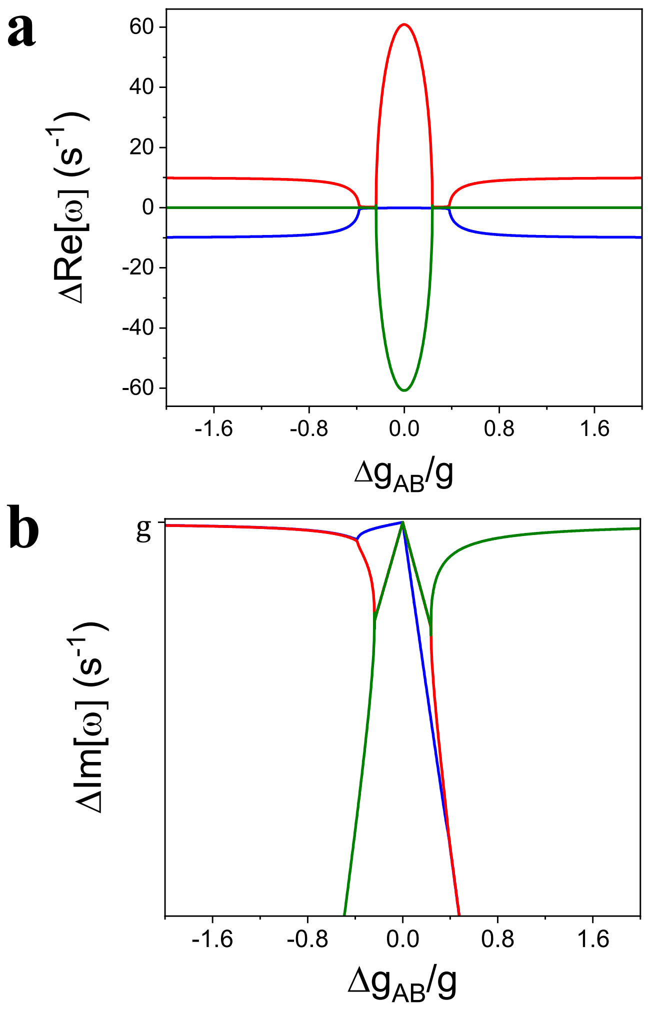

Supplementary Figure 11 Eigenfrequencies over the full range of parameter space.

a Real and **b** Imaginary part of the eigenfrequencies vs. the gain differential Δg_{AB} for κ = 3.0 x 10^{11} s^{-1}, γ_{23} = 1.8 x 10^{12} s^{-1} and γ_{13} = 0 s^{-1} The behavior is symmetric in the real frequency

splitting, whereas only mode 3 will lase while modes 1 and 2 become lossy at large positive Δg_{AB}, as expected.

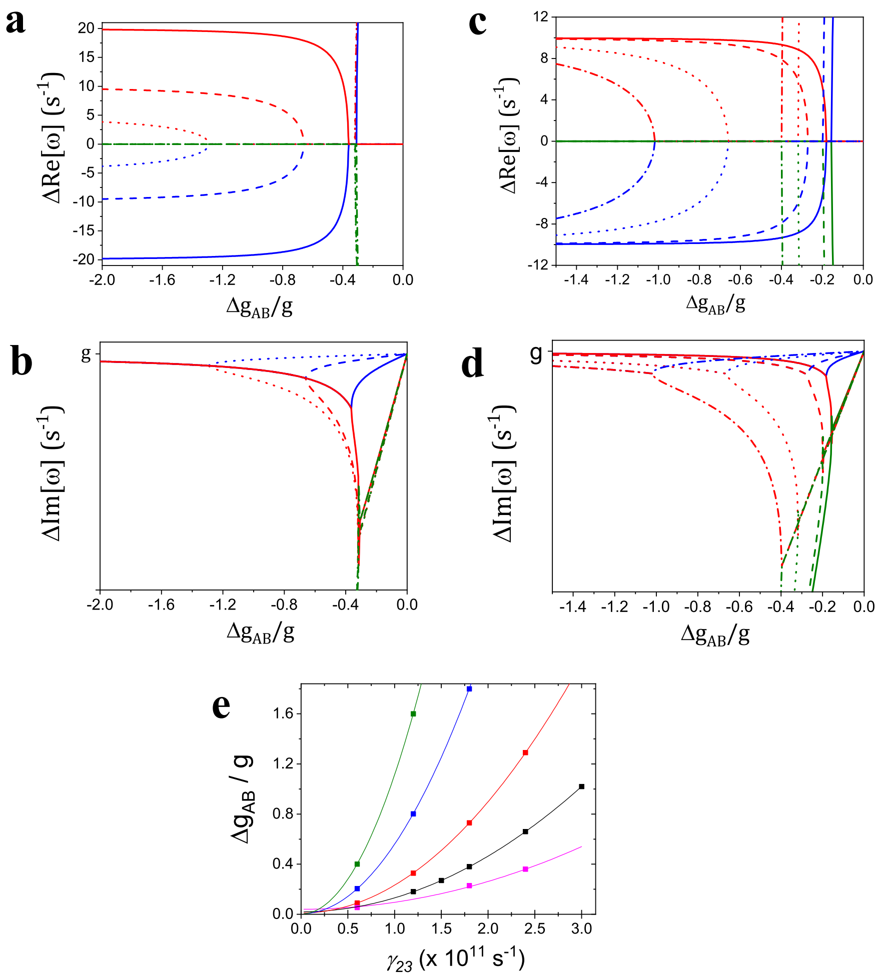

Supplementary Figure 12 Dependence of the EPs on the coupling parameters

a,b Real and imaginary part of the eigen frequencies ω_1 (blue), ω_2 (red), and ω_3 (green), vs. the gain differential Δg_{AB} for different values of κ with γ_{23} = 2.3 x 10^{11} s^{-1} and γ_{13} = 0 s^{-1}. The values of κ are 1.5 x 10^{11} s^{-1} (dotted) 3.0 x 10^{11} s^{-1} (dashed) and 6.0 x 10^{11} s^{-1} (solid) **c,d** Same as in (**a,b**) but for different values of γ_{23} with κ = 3.0 x 10^{11} s^{-1}. The values of γ_{23} are 4.5 x 10^{11} s^{-1} (solid), 6.0 x 10^{11} s^{-1} (dashed), 9.0 x 10^{11} s^{-1} (dotted) and 1.2 x 10^{12} s^{-1} (dash-dotted). **(e)** Point in Δg_{AB} where EP1 occurs vs γ_{23} for different values of κ. The solid lines are the family of solutions of the form $\Delta g_{AB} = [\kappa + (\gamma_{23})^2/2\kappa]$. The values of κ are 3.0 x 10^{10} s^{-1} (green), 6.0 x 10^{10} s^{-1} (blue), 1.5 x 10^{11} s^{-1} (red), 3.0 x 10^{11} s^{-1} (black), and 6.0 x 10^{11} s^{-1} (magenta).

Here we show the variation of the eigenvalue dynamics with different parameter values for the 3-mode Hamiltonian. In panels a,b we see the effect of varying the intra-cavity coupling, κ. The main effect is to determine the splitting at large (negative) gain differential. Increasing κ also moves the location of EP1 closer to the center. This is because the larger the coupling between the intracavity modes 1 and 2 the smaller the gain differential between microdisks A and B that is need to reach the delocalized interacting regime. Note, however, that the location of EP1 is unaffected by the value of κ. In panels c,d we see similar behavior as γ_{23} but with the opposite trend: larger coupling pushes both EP1 and EP2 farther out from the center. In panel e we summarize the dependence of the location of EP1 on κ and $\gamma_{23,}$ where conformation to the relation $\Delta g_{AB} = [\kappa + (\gamma_{23})^2/2\kappa]$ is evident. We note that if EP1 collides with EP2, intra-cavity mode coalescence cannot be reached.

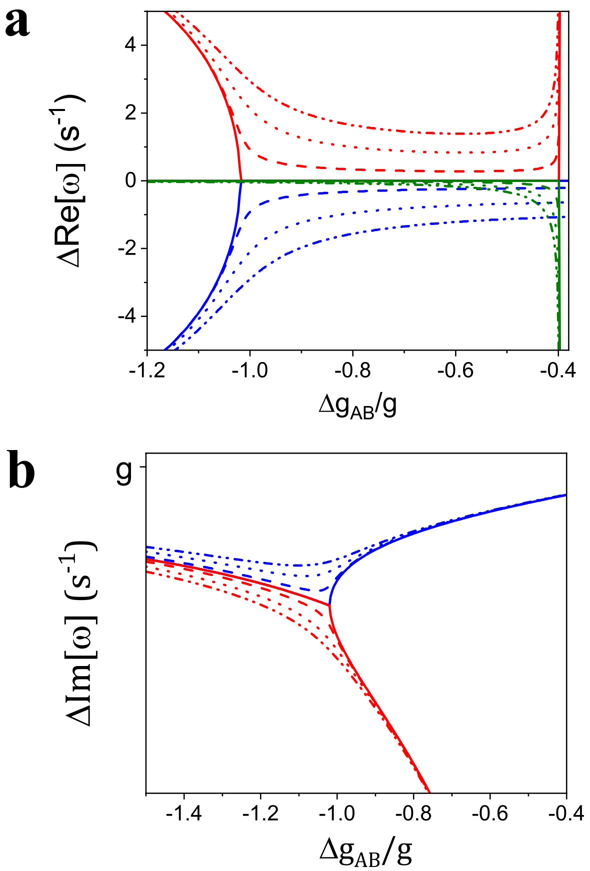

Supplementary Figure 13 Influence of the weak coupling parameter

a Real and **b** Imaginary parts of the eigenfrequencies vs. Δg_{AB} for different values of γ_{13} with both $\gamma_{23} = 3.0 \times 10^{12}$ s^{-1} and $\kappa = 3.0 \times 10^{11}$ s^{-1}. The values of γ_{13} are 0 s^{-1} (solid), 3.0×10^{10} s^{-1} (dashed), 9.0×10^{10} s^{-1} (dotted) and 1.5×10^{11} s^{-1} (dash-dot-dotted).

In Supplementary Fig. 13 we show the eigenvalue dependence on the smaller coupling term γ_{13}. It is clear that as γ_{13} increases the coalescence is transformed into and avoided crossing. In the high γ_{23} limit, the minimum splitting is $\gamma_{13}/2$. Thus, when $\gamma_{13} = 0$, a true exceptional point is obtained. It is also clear from this figure that the locations of EP1 and EP2 are independent of γ_{13}.

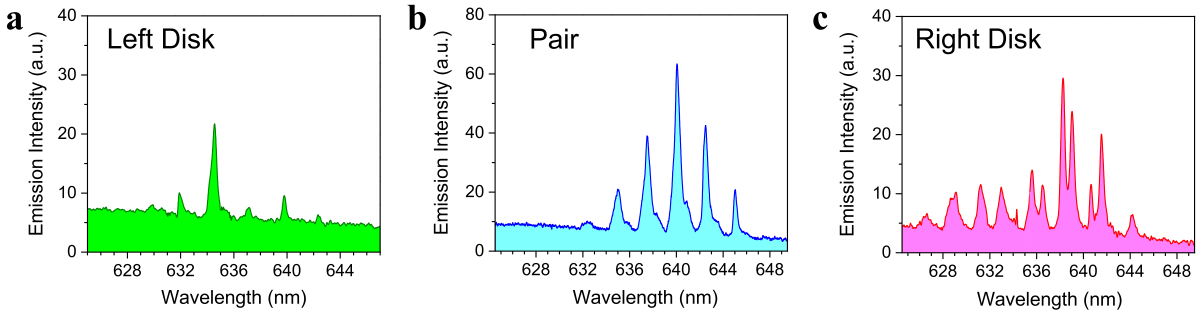

Supplementary Figure 14 Mode suppression in the asymmetric pumping scheme Laser emission spectra from a coupled microdisk pair, where the pair is placed at different locations in the pump beam spot, such that only the left **a** or right **c** microdisk is pumped, or the pair is pumped evenly **b**. The left microdisk of the pair shows significant suppression of the side modes compared to the evenly pumped pair or the right disk with substantial mode splitting due to the presence of defects.

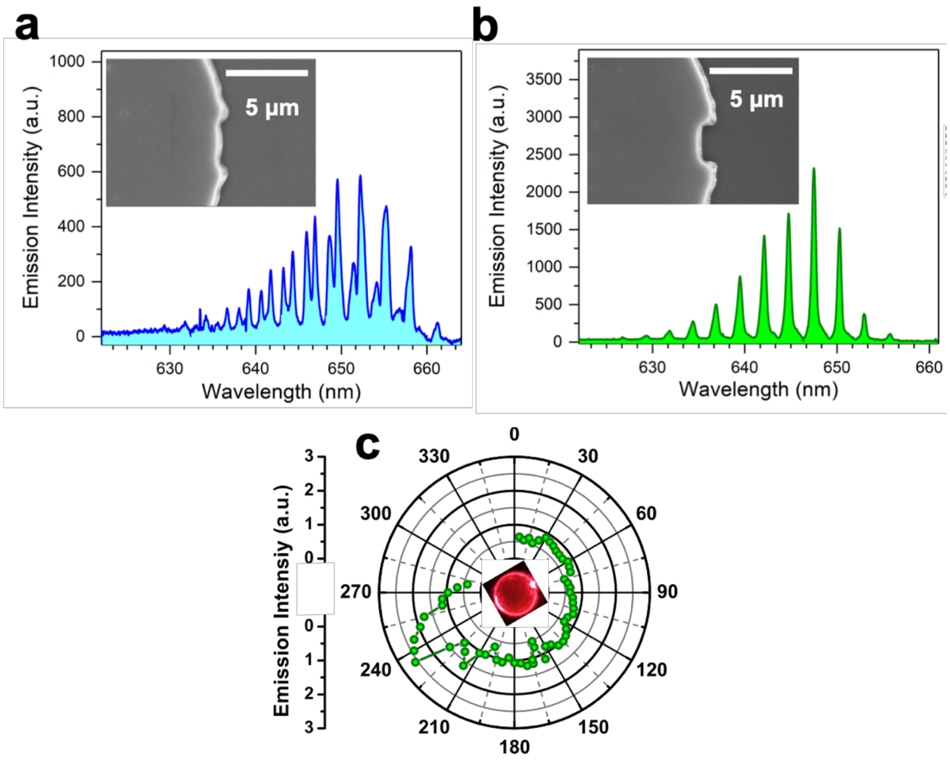

Supplementary Figure 15 Designed defect by synthetic notch implementation

a Emission spectra from a disk with a small synthetic notch implant with a radial depth of 200nm. The large degree of mode splitting is apparent. **b** Emission spectra from a disk with a large synthetic notch implant with a radial depth of 800nm. The mode-splitting is entirely

absent. **c** Collection-angle dependence of the emission in the plane of the microdisk with large synthetic notch as in **(b)** with fluorescent image at the center.

In Supplementary Fig. 15a, we show the spectrum from a resonator with a small synthetic notch implant implemented via incorporation of the structure in the photo-lithographic mask. The strong mode splitting in the emission spectrum is evident. We have further found that when the size of the notch is large, as in Supplementary Fig. 15b, the splitting is removed. Following similar work involving nano-particle scattering induced mode-splitting[2], the removal of mode-splitting by a large notch is expected due to increased loss applied selectively to one of the broken-degeneracy modes. In Supplementary Fig. 15c, we show that the notch leads to highly directional light scattering, similar to the defects in Figure 1e. These notches act as scatterers in a similar manner as the uncontrolled defects in the samples described in the manuscript, which couples the CW and CCW modes via scattering into one another, thus generating a mutual dependency of their amplitudes.

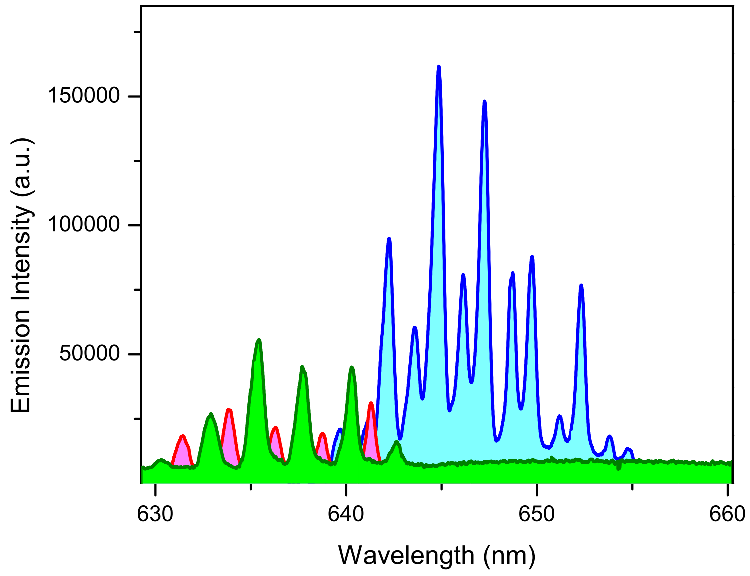

Supplementary Figure 16 Emission spectra from coupled microdisks with large synthetic notches

When the pair of coupled microdisks with large (800nm) synthetic notches are pumped in the asymmetric pumping condition (green and red), no mode splitting is observed. When the pair is pumped evenly (blue), mode splitting is observed, thus, the expected behavior is recovered[3]. The

presence of splitting also validates the evanescent coupling of microdisk separated by a distance of 400nm. From the splitting we obtain a coupling strength of ~8.0 x 10^{11} s^{-1}, which is notably higher than that obtained from the FDTD simulations described in Supplementary Fig. 6, and consistent with the values necessary to drive the coalescence at EP1. Moreover, these results suggest the role of intra-cavity coupling in obscuring the inter-cavity interaction in the presence of gain/loss contrast variation.

Supplementary References

1. Little, B. E., Chu, S. T., Haus, H. A., Forsei, J., & Laine, J.-P., Microring Resonator Channel Dropping Filters, *J. Lightwave Technol.*, **15**, 998-1005 (1997)
2. Zhu, J., Özdemir, S. K., He, L., & Yang, Y., Controlled manipulation of mode splitting in an optical microcavity by two Rayleigh scatterers, *Opt. Express*, **18**, 23535-23543 (2010).
3. Hodaei, H., Miri, M.-A., Heinrich, M., Christodoulides, D. N., & Khajavikhan, M, Parity-time-symmetric microring lasers, *Science,* **346**, 975-978 (2014).
