# Supplementary material for: Robust lasing modes in coupled colloidal quantum dot microdisk pairs using a non-Hermitian exceptional point
Source: Nat Commun. 2019 Feb 4;10:561. doi: 10.1038/s41467-019-08432-6 (PMC6362135; doi:10.1038/s41467-019-08432-6)
Supplement: Supplementary file 3 — Lasing Reporting Summary [file 41467_2019_8432_MOESM3_ESM.pdf]

nature research

Corresponding author(s): Z. Valy Vardeny

Lasing Reporting Summary

Nature Research wishes to improve the reproducibility of the work that we publish. This form is intended for publication with all accepted papers reporting claims of lasing and provides structure for consistency and transparency in reporting. Some list items might not apply to an individual manuscript, but all fields must be completed for clarity.

For further information on Nature Research policies, including our data availability policy, see Authors & Referees.

Experimental design

Please check: are the following details reported in the manuscript?

1. Threshold

Plots of device output power versus pump power over a wide range of values indicating a clear threshold

- [x] Yes
- [ ] No

Supplementary Figure 2 shows the output power threshold.

2. Linewidth narrowing

Plots of spectral power density for the emission at pump powers below, around, and above the lasing threshold, indicating a clear linewidth narrowing at threshold

- [x] Yes
- [ ] No

Supplementary Figure 2 shows the cavity modes occur at the threshold power.

Resolution of the spectrometer used to make spectral measurements

- [x] Yes
- [ ] No

The resolution of the spectrometer is stated in the Methods section. It is lower than the linewidth of modes.

3. Coherent emission

Measurements of the coherence and/or polarization of the emission

- [ ] Yes
- [x] No

Our confocal, micro-PL experimental setup does not allow determination of coherence properties or polarization information.

4. Beam spatial profile

Image and/or measurement of the spatial shape and profile of the emission, showing a well-defined beam above threshold

- [ ] Yes
- [x] No

The emission profile is shown in Figures 1d and 1f, but the narrowing of beam divergence is not measurable in the experimental setup.

5. Operating conditions

Description of the laser and pumping conditions
Continuous-wave, pulsed, temperature of operation

- [x] Yes
- [ ] No

The pumping conditions are described in the Methods section.

Threshold values provided as density values (e.g. W cm^{-2} or J cm^{-2}) taking into account the area of the device

- [x] Yes
- [ ] No

The threshold values are described in units of micro-Joules per centimeter-squared in Figure 1 and the related discussion in the Results section.

6. Alternative explanations

Reasoning as to why alternative explanations have been ruled out as responsible for the emission characteristics
e.g. amplified spontaneous, directional scattering; modification of fluorescence spectrum by the cavity

- [ ] Yes
- [x] No

The focus of the paper is on modal properties and does not depend on whether the modes are laser modes or cavity-modified amplified spontaneous emission modes. The nonlinear threshold power behavior rules out cavity-modified fluorescence.

7. Theoretical analysis

Theoretical analysis that ensures that the experimental values measured are realistic and reasonable
e.g. laser threshold, linewidth, cavity gain-loss, efficiency

- [ ] Yes
- [x] No

The experimental values of lasing properties are consistent with similar colloidal materials. Detailed theoretical analysis of our specific gain medium and cavity design are not available.

8. Statistics

Number of devices fabricated and tested

- [x] Yes
- [ ] No

Statistical information is provided in Figure 2g and Supplementary Figures 8 and 9.

Statistical analysis of the device performance and lifetime (time to failure)

- [ ] Yes
- [x] No

The emission properties are stable and described in Reference 30.
